# Evaluation methods of artificial demineralization protocols for coronal dentin: a systematic review of laboratory studies

**DOI:** 10.1186/s12903-025-05885-8

**Published:** 2025-04-24

**Authors:** Alaa Esmat Abdel Salam, Hoda Saleh Ismail, Hamdi Hamama

**Affiliations:** 1 Conservative Dentistry Department, Faculty of Dentistry, Mansoura University, Mansoura, Egypt; 2 Faculty of Dentistry, New-Mansoura University, New-Mansoura City, Egypt

**Keywords:** Artificial caries model, Chemical model, PH-cycling, Dentin mineral density, Depth of lesion, Elemental analysis

## Abstract

**Background:**

Despite the widespread use of various laboratory models such as chemical, bacterial, or combination as demineralization protocols to induce artificial caries in dentin, there is lack in the literature regarding a comparison of their effectiveness and assessment of dentin mineral density through using several analytical techniques, including microscopic and spectroscopic ones. The purpose of this review was to determine the appropriate demineralization protocols for inducing artificial caries utilizing coronal dentin. Furthermore, this evidence-based study was conducted to identify the most reliable evaluation methods in assessing the efficiency of the reviewed protocols.

**Materials and methods:**

An electronic search was conducted on three databases: MEDLINE/PubMed, Scopus, and ScienceDirect, following PRISMA guidelines. Only the studies published between 2019 and 2024 were considered. All studies were assessed based on predefined eligibility criteria. English laboratory studies that employed chemical models for induction of artificial caries on human mid-coronal dentin were included. The selected studies were individually reviewed for potential bias according to predetermined criteria.

**Results:**

A total of 23 studies met the inclusion and exclusion criteria of this systematic review. From the included studies, 11 studies utilized pH-cycling model, 10 studies reported using simple demineralization model, while only one study reported an undetailed protocol. Moreover, combined ‘chemical and biological’ protocol for dentin demineralization was reported in two studies. According to the outcome of included studies, dentin mineral density, depth of lesions, crystalline structure, surface morphology, and surface microhardness were evaluated using a variety of laboratory methods offering, either qualitative, quantitative, or semi-quantitative analysis. The conclusions of the studies revealed confirmatory results regarding the use of multiple devices.

**Conclusions:**

The pH-cycling model was found to be the most common type of chemical model used to induce dentin demineralization for 14 days immersion time, followed by a simple demineralization model through using an acetic acid solution. There was no single evaluation approach found to provide comprehensive information about the mineral content independently. Therefore, a combination of multiple techniques is recommended to yield sufficient and more accurate data.

**Supplementary Information:**

The online version contains supplementary material available at 10.1186/s12903-025-05885-8.

## Background

Dental caries is the most prevalent chronic disease that involves localized destruction of dental hard tissues [1, 2]. This complex process occurs over time through interaction between acid-producing bacteria and fermentable carbohydrates, influenced by various host factors such as the teeth and saliva [3]. The caries procedure that took place in dentin is considered more complicated than enamel [4, 5]. This might be attributed to the heterogeneous structure of dentin and the reactive changes of such dynamic tooth substrate [5]. Dental caries can induce some clinically significant alterations in dentin substrate, ranging between soft, firm/leathery, and hard dentin caries [6, 7]. Complete caries removal reaching the hard dentin is no longer used, however, selective caries removal to soft dentin is highly recommended [7].

Caries-affected dentin is a critical substrate, which was comprehensively evaluated in studies that focused on demineralization/remineralization procedures, prevention of caries progression, removal of caries, and restorative materials bonding to dentin [8]. Such a preservable type of dentin exhibits distinct alteration in its organic ‘collagen fibers’ and abundant loss of its ‘inorganic’ content, additionally, it shows a minimal degree of demineralization, high water content with relatively intact collagen fibers [9, 10]. Despite significant advancements in adhesion over recent decades, bonding to caries-affected dentin presents a challenging substrate during restorative procedures. Consequently, it remains an important topic, as several studies revealed that the bond strength to such substrate is lower than that of sound dentin [11]. In order to overcome the challenges of a lack of standardization associated with using natural caries-affected dentin, artificial caries methods have been developed to create a standardized carious lesions that mimic natural caries-affected dentin [12]. Researchers have employed artificial caries models to test methods for caries removal, particularly to evaluate the adhesion to selectively removed carious dentin [11, 13, 14].

**Fig. 1:**
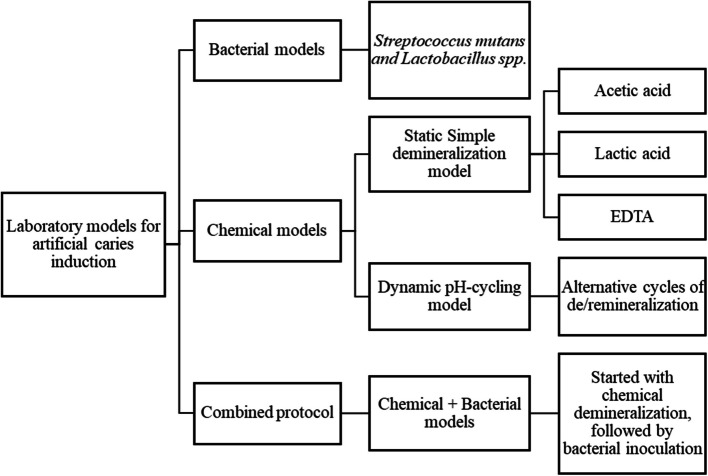
Classification of laboratory models for artificial caries induction

Over the past few decades, various laboratory caries models have been developed for induction of artificial caries to evaluate the dental caries process and assess the efficacy of remineralizing agents on demineralized dentin [15]. This is attributed to difficulties and high expenses of conducting clinical trials, which in some cases cannot be conducted due to ethical considerations [16]. Laboratory models are characterized by their simplicity and low-cost, allowing for reproducible experiments in a controlled and simplified manner [17]. Nevertheless, conducting some studies under strict standardized conditions, authors reported that achieving consistent and reproducible lesions is too challenging due to influence of various factors on the demineralization process [18]. Classification of concurrent laboratory models for induction of artificial caries is presented in Fig. 1 [17].

The characteristics of resultant artificial carious lesions, such as lesion depth, mineral loss, and mineral distribution, are affected by modifying factors like the type of demineralizing solution, its concentration, pH value, immersion time, temperature, and the presence of mineral dissolution inhibitors [19]. It is believed that artificial carious lesions which induced by chemical models provide a morphological simulation and similar hardness values to natural lesions [20]. According to Moron et al*.* [21] who emphasized the difficulties in the standardization of lesion development due to the complex interactions between demineralizing agents and dentin tissues. Additionally, Azevedo et al*.* [12] evaluated a protocol for obtaining standard CAD using a *Streptococcus mutans* biofilm for the demineralization process. The outcome of this study demonstrated that achieving uniform lesion characteristics is complicated due to the influence of various factors during the demineralization procedure.

The extent of demineralization and remineralization processes in dentin has been widely studied through the development of advanced microscopic and spectroscopic techniques [22]. Laboratory microscopic techniques can qualitatively evaluate the surface morphology. On the other hand, spectroscopic evaluation methods which involve generation and interpretation of spectra in order to provide valuable information about the mineral content, chemical composition, and depth of lesion [23, 24]. These methods could be classified as either destructive or non-destructive methods. The first type requires specific specimen preparation that might include coating, desiccation, vacuuming, polishing, or tooth sectioning, which may destroy the surface and impair the analysis. While non-destructive method allows repeated measurements to same specimens and an eco-friendly testing method [25].

In the cariology research, it was found that several laboratory studies utilized demineralization protocols for induction of artificial caries to dentin, they addressed some variations in outcomes and standardization methods [8, 11, 21, 26]. However, they highlighted various changes taking place in dentin ranging from massive mineral loss to sclerosing dentin changes, which occur in certain circumstances of slowly-progressing carious lesions [27]. These discrepancies emphasize the need for standardized methodologies to ensure reproducibility and comparability across studies. Therefore, there is a significant research gap regarding a comprehensive comparison of the effectiveness and outcomes of dentin demineralizing protocols, paired with the diverse analytical methods employed to assess dentin mineral density. Furthermore, the lack of recent reviews that systematically assess the different evaluation methods for measuring dentin mineral density emphasizes the significance of this research. Thus, it is important to have summarized data, based on a systematic search of the scientific literature.

In light of previously mentioned research gaps, the primary aim of this systematic review was to answer the research questions, aiding researchers in selecting the appropriate demineralization protocol for inducing artificial caries in mid-coronal dentin. This involved some considerations such as the type of solution, its concentration, pH value, immersion time, and temperature. Additionally, this review aimed to investigate the suitable evaluation procedures through highlighting their strengths and limitations for assessing the efficacy of demineralization protocols and determining various parameters such as dentin mineral density, lesion depth, elemental analysis, and surface morphological analysis.

## Materials and methods

### Information source and systematic search

The protocol of this systematic review was designed following Preferred Reporting Items for Systematic Reviews and Meta-Analysis (PRISMA) guidelines [28]. This protocol can be accessed through the PROSPERO registration number (CRD42024587504).

**Fig. 2:**
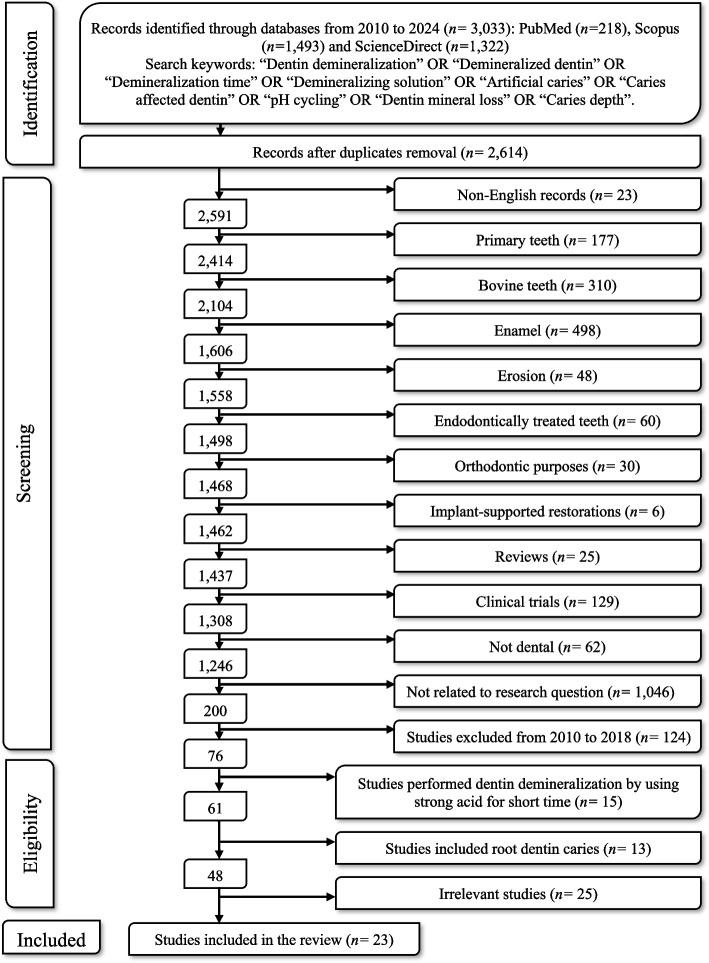
Study flowchart as adapted from the PRISMA Statement

An electronic search was performed via three databases: National Library of Medicine (MEDLINE/PubMed), Scopus, and ScienceDirect. However, only studies published between 2019 and 2024 were included in the search. This is illustrated in the flowchart (Fig. 2). The following keywords were used to search on the previously mentioned databases: “Dentin demineralization” OR “Demineralized dentin” OR “Demineralization time” OR “Demineralizing solution” OR “Artificial caries” OR “Caries affected dentin” OR “pH cycling” OR “Dentin mineral loss” OR “Caries depth”. Furthermore, a subsequent manual search was conducted to check for non-online resources. Then the selected articles were imported to EndNote 21 software (Thompson Reuters, Philadelphia, PA, USA) to remove duplicates. A gray literature search was conducted following the online database search.

### The PICO framework

Referring to the (PICO) framework, population (P), intervention (I) and outcome (O), the following PICO questions were established:

- **Population:** artificial caries model was performed in sound coronal dentin.
- **Intervention:** demineralization protocol was done through immersion procedure of the specimens in demineralizing agents for a specific time.
- **Outcome:** changes in mineral content in dentin.

Therefore, the key questions of this systematic review were: (1) What is the proper demineralization protocol to create artificial caries in mid-coronal human dentin including the type of demineralizing agents and the proper demineralization time? (2) What is the proper evaluation procedure for determination of dentin mineral density after dentin demineralization in an artificial caries model?

### Eligibility criteria (Inclusion/exclusion criteria)

The collected studies were assessed following the inclusion criteria: studies published from 2019 to 2024, English-written manuscripts, laboratory studies including artificial caries model on human mid-coronal dentin, studies related to dentin demineralization through pH-cycling, different demineralizing agents, different time of immersion, different techniques to evaluate dentin mineral loss, caries-affected dentin, chemical and combined (chemical and microbial) models for dentin demineralization.

After evaluating the studies according to these inclusion criteria, the following studies were excluded: non-English manuscripts, clinical studies, studies using primary teeth or animal teeth, review articles, clinical trials, case reports, studies related to endodontics or orthodontics or implantology, studies performed on enamel, studies related to dentin erosion, studies not related to Dentistry or not being related to research question, studies published before 2019. Moreover, studies which performed on root dentin or utilized strong acid (e.g. phosphoric acid) for a short time were also excluded.

### Search strategy

After removal of duplicates, all studies were assessed according to the eligibility criteria. Assessment of studies went through 3 stages: (1) assessment according to the title, (2) abstract, and (3) analysis for studies’ full text. All studies that were electronically and manually searched were collected, printed, and distributed among all authors. The eligibility criteria were checked by each author for all included studies. The agreement of at least two authors was required for study inclusion.

### Assessment of risk of bias

Critical Risk Assessment of in-vitro Studies (CRIS) tool was used to perform the quality assessment of the included studies. The risk of bias was independently assessed by A.E. and displayed the results to the second reviewer (H.S.), using parameters employed in previous systematic reviews [19]. If a parameter was reported in the study, it was assigned a"yes,"indicating its use. If a parameter was not reported, it was assigned a"no."Studies reporting 1 or 2 parameters were classified as having a high risk of bias, those reporting 3 parameters were categorized as having a medium risk of bias, and studies reporting 4 or 5 parameters were considered to have a low risk of bias.

Bias risk was assessed based on five parameters: utilization of sound/healthy/caries-free teeth, creation of a smear layer, randomization of groups and subgroups, inclusion of a control group, and examiner blinding regarding testing equipment. RevMan 5.4 (RevMan 5.4, The Cochrane Collaboration, Copenhagen, Denmark) was employed to generate a summary and graphical representation of bias risk for the chosen studies.

## Results

### Search results

The initial search of the 3 databases; PubMed (218 studies), Scopus (1,493 studies), and ScienceDirect (1,322 studies), resulted in a total number of 3,033 studies. Firstly, duplicate records (419) were removed, followed by the exclusion of non-English records (23). At the title/abstract level, 2,507 records were excluded based on the predefined criteria. Specifically, 177 studies were excluded for using primary teeth, 310 studies for using bovine teeth, 498 studies related to enamel, 48 studies related to dentin erosion, 60 studies for using endodontically treated teeth, 30 studies for orthodontic purposes, 6 studies for implant-supported restorations, 25 studies for reviews, and 129 studies for clinical trials. Additionally, 62 studies were excluded as they were unrelated to dentistry, and 1,046 studies were not relevant to the research question. Furthermore, 124 studies published before 2019 were excluded.

Full-text articles were assessed for eligibility, resulting in 76 studies. Among them, 13 studies were excluded due to the use of root dentin, and 15 studies were excluded due to the use of strong acid for a short time to simulate dentin etching. The remaining 25 studies were deemed irrelevant. Ultimately, 23 studies met the inclusion criteria for this systematic review. The search process stages are depicted in the flowchart (Fig. 2).

### Data extraction

This systematic review evaluated 23 studies [26, 29–50] that employed various chemical demineralization protocols to simulate artificial caries in coronal human dentin. The included studies investigated the type of demineralizing agent, proper demineralization time, and different evaluation procedures for determining dentin mineral density or the depth of dentinal lesion in artificial caries models. The extracted data from the studies are summarized in Table 1.

**Table 1:** Scientific studies, aim, and conclusion

Author(s)/Year	Aim of the study	Conclusion
Francois et al*. * [29] (2024)	Evaluation of remineralization ability of various ion-releasing materials on artificially demineralized dentin	Cention Forte showed significant increase in the MGVs of artificially demineralized dentin while EQUIA Forte HT showed no significant MGV increase
Aruna Rani et al*.* [30] (2023)	Assessment of remineralization efficacy of CEnHAp with and without PHS on artificially induced dentin lesions under pH-cycling	CEnHAp-PHS inhibits demineralization and promotes remineralization on demineralized dentin so it can be considered as a potential dentin remineralizing agent
Cifuentes-Jiménez et al*.* [31] (2023)	Evaluation of the remineralizing capacity of SDF/NaF on demineralized dentin	The use of SDF and 0.2% NaF solution has a remineralizing effect on demineralized dentin
Fernandes et al*.* [32] (2023)	Investigation of pulp cells response with an odontoblastic phenotype to SDF and KI application on demineralized dentin	Treatment of demineralized dentin with 38% SDF presents a mild to moderate toxic effect on pulp cells which prevented by applying KI
Rao et al. [33] (2023)	Evaluate the remineralization of artificially induced CAD when treated with universal adhesive modified with (PAMAM) loaded with MBG and its effect on μTBS	The complex of PAMAM-MBG-Universal adhesive can remineralize the demineralized CAD thereby improving its bond strength
Aldosari and Al-Sehaibany [34] (2022)	To investigate the effect of loading time on the color stability of RBC, RMGIC, and GIC bonded to SDF-treated demineralized dentin	Delayed loading time of the restorative material for one week following SDF application resulted in greater color stability than that of immediate loading
Khor et al*.* [35] (2022)	To determine if SDF application to sound and ACAD immediately prior to GIC restoration affected μTBS	SDF application immediately prior to GIC resulted in a significantly lower μTBS on sound dentin but had no significant effect when applied to ACAD
QI et al*.* [36] (2022)	To evaluate the effects of helium CAP on the bonding performance and surface modification to CAD	The helium CAP jet treatments for 30 s and 45 s are effective in improving the immediate bond strength of CAD and slows the aging process of the bonding
Silva et al*.* [37] (2022)	Evaluation of the bond strength and adhesive interface characteristics of sound, NCAD, and ACAD under SPP and after aging in solvents	The types of dentin, aging solvents, and storage time negatively influenced the bond strength under SPP
Steier et al*.* [38] (2022)	Evaluation the accuracy of caries detection and the application-sensitivity of the new designs for Vision’s REVEAL™ utilizing a fluorescence activating headlight for excitation purposes	Fluorescent enhanced theragnosis through Reveal vision glasses can ensure constant monitoring and diagnosis of caries progress for a better outcome
Babaie et al*.* [39] (2021)	Evaluate the efficacy to remineralize artificial and natural dentin lesions through restorative procedures that include PILP method comprising pAsp.	The findings indicate the benefit of PILP applications in the functional repair of dentin caries and illustrate the integration of PILP-method into a restorative approach in minimally invasive dental procedures
Dai et al. [40] (2021)	To investigate the remineralizing effect of a HX-BGC and fluoride on demineralized enamel and dentin	Combined use of HX-BGC and fluoride can reduce mineral loss and promote remineralization of demineralized enamel and dentine
Abdelshafi et al. [41] (2021)	Evaluate the application of synthesized Col/Hap nanocomposite with collagen cross-linker to improve the quality of DRCZ structure and increase its bond strength to adhesive resin materials	The applied Col/Hap nanocomposites with higher Hap and lower Col had the most significant impact to increase μTBS
Cifuentes-Jimenez et al*.* [42] (2021)	Investigate the effect of SDF agents on the chemical composition and microstructural properties of dentin, and its relation to the bond strength of the adhesives	SDF agents resulted in formation of crystalline phases of silver salts and increased mineralization of demineralized dentin. SDF application showed a negative effect on bond strength of the adhesives
Sami et al*.* [43] (2021)	Assessment of the bonding effect to CAD on the shear bond strength of two universal adhesives applied in different adhesion protocols compared with sound dentin	Single Bond universal adhesive in an etch-and-rinse adhesion protocol improved only bonding to sound dentin, while a negative effect was found for the etching step with Prime and Bond universal adhesive when bonded to both sound and CAD substrates
Chen et al*.* [44] (2020)	Assessment of the ultrastructural change of demineralized dentin collagen during Ca/P-PILP remineralization process, evaluation of the remineralization potential of Ca/P-PILP at demineralized ACAD and investigation of the bond integrity and bond strength of the remineralized ACAD	Ca/P-PILP induces remineralization of demineralized dentin collagen. ACAD can be partially remineralized by using high concentration Ca/P-PILP. Besides, the bonding integrity of remineralized ACAD can be improved and the bonding strength can be significantly increased
Daneshpoor and Pishevar [45] (2020)	Evaluation of bioactivity and remineralizing ability of calcium silicate cements and CPP-ACP on demineralized dentin	Bioactive cements and CPP-ACP had bioactivity capability during one week. Demineralized dentin could be remineralized with bioactive materials
Sadoon et al*.* [46] (2020)	Assessing the remineralizing ability of the pulp protecting materials in the presence and in absence of non-collagenous proteins analogs	Calcium silicate-based cement had a better remineralization potential than hydroxyapatite-based cement either in presence or absence of biomimetic analogs
Scholz et al*.* [47] (2020)	Investigate the influence of complete or selective excavation of artificial caries lesions on the marginal integrity	No significant difference was found in the marginal integrity of restorations made in teeth exposed to complete excavation, selective excavation, or caries-free control lesions
Wu et al*.* [48] (2020)	Investigate the remineralization effect of bioactive glass on artificial dentine caries	Bioactive glass had a promising remineralization effect on artificial dentine caries and could be a therapeutic choice for caries management
Zhao et al*.* [49] (2019)	Investigate the effect of SDF and KI treatment on tooth discoloration and the shear bond strength of GICs to ACAD	Immediate application of KI after SDF treatment can reduce dentin discoloration caused by SDF. Furthermore, SDF + KI treatment does not negatively affect bonding of GICs to ACAD
Schwendicke et al. [26] (2019)	Evaluate the remineralization effects of ion-releasing materials in chemically or bacterially-induced dentin caries lesions	Biodentine and MTA induced mineral precipitation, but intra/inter-fibrillar collagen mineral infiltration was only provided by biomimetic remineralization via the use of the experimental adhesive
Saxena et al*.* [50] (2019)	Evaluate the remineralization of demineralized dentin via dual analog system using a TPP “templating analog” and a PAA or pAsp “sequestration analog”	PAA was unable to remineralize demineralized dentin even when pre-treated with TPP. However, pre-treatment with TPP enhanced mineralization of specimens that were PILP-remineralized using pAsp.

*Abbreviations*: *MGVs* Mean gray values, *CEnHAp* Chicken eggshell–derived nanohydroxyapatite, *PHS* Phytosphingosine, *SDF* Silver diamine fluoride, *CAD* Caries-affected dentin, *PAMAM* poly(amidoamine) dendrimer, *MBG* Mesoporous bioactive glass nanoparticles, *μTBS* Microtensile bond strength, *RBC* Resin-based composite, *RMGIC* Resin-modified glass ionomer cement, *GIC* Glass ionomer cement, *ACAD* Artificial caries-affected dentin, *CAP* Cold atmospheric plasma, *NCAD* Natural caries-affected dentin, *SPP* Simulated pulpal pressure, *PILP* Polymer-induced liquid precursor, *pAsp* polyaspartic acid, *HX-BGC* strontium-doped bioactive glass, *Col/Hap* Collagen/hydroxyapatite, *DRCZ* Deeper and remineralizable carious zone, *Ca/P-PILP* Calcium phosphate polymer-induced liquid precursor, *CPP-ACP* Casein phosphopeptide-amorphous calcium phosphate, *MTA* Mineral trioxide aggregate, *TPP* Tripolyphosphate, *PAA* Poly acrylic acid

### Descriptive analysis

All included studies used chemical models for artificial dentin caries induction except two studies; the first study [38] used only the combined protocol (chemical and biological model) for dentin demineralization, while the second one [26] evaluated both demineralization protocols (chemical model and combined protocol). The details of chemical models are illustrated in Table 2.

**Table 2:** Different types of chemical models used for artificial caries induction

Author(s) /Year	De-mineralizing protocol	Demineralizing Solution	pH	Remineralizing solution	pH	Time/amount of solution	Days	Temperature
Francois et al*.* [29] (2024)	pH-cycling model	2.2 mM CaCl_2_, 2.2 mM NaH_2_PO_4_ and 50 mM acetic acid	3.5	1.5 mM CaCl_2_, 0.9 mM NaH_2_PO_4_, and 0.15 M KCl	7.1	Each group was immersed in 100 mL of demineralizing solution for 8 h and in remineralizing solution for 16 h	14	Room temperature
Aruna Rani et al*.* [30] (2023)	Simple de-mineralization model	2 mM CaCl_2_.2H_2_O, 0.0476 mM NaF, 2.2 mM KH_2_PO_4_, 50 mM acetic acid and 10 mM KOH	5	-	-	The dentin slabs were immersed in (20 ml/slab) of demineralizing solution	5	37 °C
Cifuentes-Jiménez et al*.* [31] (2023)	pH-cycling model	2.2 mM CaCl_2_, 2.2 mM NaH_2_PO_4_, and 50 mM acetic acid	4.8	1.5 mM CaCl_2_, 0.9 mM NaH_2_PO_4_, and 0.15 M KCl	7	Specimens were immersed in 1 ml of a demineralizing solution for 8 h and for 16 h in 1 ml of a remineralizing solution	14	Room temperature
Fernandes et al*.* [32] (2023)	Simple de-mineralization model	2.2 mmol/L of CaCl_2_, 2.2 mmol/L of NaH_2_PO_4_, and 50 mmol/L of acetic acid	4.5	-	-	Specimens received 300 μL of an acidic solution	2	Room temperature (25 °C)
Rao et al. [33] (2023)	pH-cycling model	2.2 mM CaCl_2_, 2.2 mM NaH_2_PO_4_, 0.05nM acetic acid	4.5	1.5 mM CaCl_2_, 0.9 mM NaH_2_PO_4_, 0.15 mM KCl	7	Specimens were immersed in 10 ml of demineralizing solution for 8 h and in 10 ml of remineralizing solution for 16 h	14	Room temperature
Aldosari and Al-Sehaibany [34] (2022)	Simple de-mineralization model	0.1 M lactic acid, 4.1 mM Ca (as CaCl_2_ × 2 H_2_O), 8 mM PO_4_ (as KH_2_PO_4_) and 0.2% w/v Carbopol 907, sodium azide 3 mM was added as bacteriostat	5	-	-	Specimens were demineralized in 10 ml demineralizing solution per specimen	7	37 °C
Khor et al*.* [35] (2022)	pH-cycling model	2.2 mM CaCl_2_, 2.2 mM NaH_2_PO_4_, and 50 mM acetic acid	4.5	1.5 mM CaCl_2_, 0.9 mM NaH_2_PO_4_ and 0.15 M KCl	7	Specimens were immersed in 10 mL of demineralizing solution for 8 h and in 10 mL of remineralizing solution for 16 h	14	-
QI et al*.* [36] (2022)	pH-cycling model	2.2 mM CaCl_2_, 2.2 mM NaH_2_PO_4_, and 50 mM acetic acid	4.5	1.5 mM CaCl_2_, 0.9 mM NaH_2_PO_4_ and 150 mM KCl	7	Specimens were suspended in demineralizing solution for 8 h and in remineralizing solution for 16 h	14	37 °C
Silva et al*.* [37] (2022)	pH-cycling model	2.2 mM CaCl_2_, 2.2 mM NaH_2_PO_4_, 0.05M sodium acetate, 0.05M acetic acid, 1 ppm fluoride (NaF)	4.5	1.5 mM CaCl_2_, 0.9 mM NaH_2_PO_4_, 0.15M KCl, 0.1M Tris buffer, 10 ppm fluoride (NaF)	7	Process included 8 demineralization/mineralization cycles. Each cycle included 3 h immersion in demineralizing solution (156.25 mL/tooth) followed by 45 h immersion in mineralizing solution (78.125 mL/tooth)	-	37 °C
Babaie et al*.* [39] (2021)	Simple de-mineralization model	0.05 M acetate buffer, 2.2 mM calcium and phosphate	4.5 5	-	-	At pH = 4.5 to create deep lesions (~ 700 μm) At pH = 5 for 66 h to create shallow lesions (~ 140 μm)	7 -	37 ◦C Room temperature
Dai et al. [40] (2021)	Demineralizing solution followed by remineralizing solution	2.2 mM CaCl_2_, 2.2 mM KH_2_PO_4_, 50 mM acetic acid	4.4	1.5 mM CaCl_2_, 0.9 mM NaH_2_PO_4_, 150 mM KCl	7	Specimens were immersed in demineralizing solution for 8 days and then in remineralizing solution for 24 h	8 1	37 ◦C
Abdelshafi et al. [41] (2021)	pH-cycling model	2.2 mmol (mM) CaCl_2_, 2.2 mM NaH_2_PO_4_, and 50 mM acetic acid	4.8	1.5 mM CaCl_2_, 0.9 mM NaH_2_PO_4_, and 0.15 M KCl	7.2	Each specimen was cycled in 10 mL of both solutions for 8 h in demineralizing and 16 h in remineralizing solutions	14	Room temperature
Cifuentes-Jimenez et al*.* [42] (2021)	pH-cycling model	2 mM CaCl_2_, 2.2 mM NaH_2_PO_4_, and 50 mM acetic acid	4.8	1.5 mM CaCl_2_, 0.9 mM NaH_2_PO_4_, and 0.15 M KCl	7	Specimens were immersed in 10 ml of a demineralizing solution for 8 h and in 10 ml of a remineralizing solution for 16 h	14	Room temperature
Sami et al*.* [43] (2021)	pH-cycling model	2.2 mM CaCl_2_, 2.2 mM NaH_2_PO_4_, 0.05 M acetic acid	4.5	1.5 mM CaCl_2_, 0.9 mM NaH_2_PO_4_, 0.15 mM KCl	7	Specimens were immersed individually in a demineralizing solution for 8 h and in a remineralizing solution for 16 h	14	-
Chen et al*.* [44] (2020)	pH-cycling model	50 mM acetic acid, 2.2 mM CaCl_2_, 2.2 mM NaH_2_PO_4_	4.8	1.5 mM CaCl_2_, 0.9 mM KH_2_PO_4_	7	Each tooth was immersed in 20 mL of demineralizing solution for 8 h followed by 20 mL of remineralizing solution for 16 h	14	-
Daneshpoor and Pishevar [45] (2020)	Simple de-mineralization model	17% EDTA	-	-	-	Specimens received 15 ml 17% EDTA for 2 h	-	Room temperature
Sadoon et al*.* [46] (2020)	pH-cycling model	2.2 mM CaCl_2_, 2.2 mM NaH_2_PO_4_, and 50 mM acetic acid	4.8	1.5 mM CaCl_2_, 0.9 mM NaH_2_PO_4_, and 0.15M KCl	7	The teeth were cycled in 150 mL of both solutions for 8 h in demineralizing solution and 16 h in remineralizing solution	14	Room temperature
Scholz et al*.* [47] (2020)	Simple de-mineralization model	0.5M Ethylenediamine-tetraacetate (EDTA Disodium Salt 2-Hydrate)	7	-	-	Each tooth was placed in 400 ml EDTA	4	Room temperature
Wu et al*.* [48] (2020)	Simple de-mineralization model	0.05M acetic acid containing 2.2 mM CaCl_2_.2H_2_O and 2.20 mM KH_2_PO_4_	5	-	-	-	3	37 °C
Zhao et al*.* [49] (2019)	Simple de-mineralization model	50 mM acetate, 2.2 mM KH_2_PO_4_, 2.2 mM CaCl_2_	4.4	-	-	-	7	25 °C
Schwendicke et al. [26] (2019)	Simple de-mineralization model	50 mM acetic acid, 3 mM CaCl_2_ × H_2_O, 3 mM KH_2_PO_2_ and 6 mM methyl-hydroxy-diphosphonate	5.3	-	-	Specimens were immersed in 5 L of a demineralizing solution for shallow lesions For deep lesions, specimens were immersed in the same acetic acid solution	14 28	37 C
Saxena et al*.* [50] (2019)	Simple de-mineralization model	0.5 mol/L EDTA in 0.5 mol/L Tris buffer	-	-	-	Dentin slices were demineralized in 1 L of demineralizing solution	6	25 °C

### Assessment of methodology

#### Type of teeth

Five studies [30, 34, 38, 43, 48] of the 23 studies utilized human premolars (22%), while the remaining included studies used human molars (78%).

#### Type of dentin

Six studies [30, 31, 33, 38, 39, 43] of the 23 studies used mid-coronal dentin (26%), while one study [26] used deep dentin (4%). The remaining included studies did not specify the type of dentin used (70%).

#### Smear layer creation

The creation of a standard smear layer was mentioned in five studies [30, 33, 36, 43, 48] (22%). It was performed by finishing the dentin surfaces with 600-grit silicon carbide paper for 60 s. One of these studies [48] used 600-grit, 800-grit, and 1200-grit silicon carbide paper on dentin surfaces. The surfaces were then ultrasonically washed in deionized water three times for 60 s each to create a uniform smear layer. The remaining studies did not mention this step (78%).

### Chemical demineralizing protocols

#### PH-cycling model

Eleven studies [29, 31, 33, 35–37, 41–44, 46] used pH-cycling models for artificial caries induction (48%). These models involved successive demineralization and remineralization cycles which mimic the dynamics of mineral loss and gain in the oral cavity. Regarding the types of de/remineralizing agents, the specimens were immersed in a demineralizing solution composed mainly of CaCl_2_, NaH_2_PO_4_, and acetic acid, followed by immersion in a remineralizing solution composed of CaCl_2_, NaH_2_PO^4^, and KCl. Other less used compounds such as NaF and Tris buffer were added to the remineralizing solution as mentioned in one of the included studies [37]. In all studies that employed pH-cycling model, specimens were immersed in demineralizing solution for 8 h followed by 16 h’ immersion in the remineralizing solution. All the included studies followed this protocol, except for one study [37] which reported demineralization/remineralization cycle; (immersing specimens for 3 h in demineralizing solution followed by 45 h immersion in a remineralizing solution).

The pH values used for the demineralizing solution varied from 3.5 to 4.8. Five studies [33, 35–37, 43] used a demineralizing solution with a pH of 4.5, another five studies [31, 41, 42, 44, 46] used a pH of 4.8, and one study [29] used a pH of 3.5. The remineralizing solution had a neutral acidity with a pH of 7 in all studies. The duration of pH-cycling was typically 14 days in all studies. Regarding the temperature, the most frequently used values were room temperature and 37 °C, however, some studies did not report the temperature. Regarding stirring or agitation, pH-cycling models were done in most of the studies without agitation, however, three studies [37, 43, 44] did not mention this step.

#### Simple demineralization model

Simple demineralization models were used in 10 studies [26, 30, 32, 34, 39, 45, 47–50] (44%), which included different types of demineralizing agents with varying immersion times, pH levels, and temperatures. Three demineralizing solutions were used; acetic, ethylenediaminetetraacetic (EDTA) or lactic acids. Six of the included studies [26, 30, 32, 39, 48, 49] used acetic acid, three utilized EDTA [45, 47, 50], and only one study [34] immerse the specimens in lactic acid, where Aldosari and Al-Sehaibany added sodium azide to lactic acid solution to act as a bacteriostatic agent.

The total immersion time of specimens in demineralizing solutions showed a high degree of variability, ranging from two to seven days. However, one study [26] reported an immersion time of 14 days to produce shallow lesions and 28 days for deep lesions. The prementioned ten studies reported variations in the pH value of the three demineralizing agents, ranging between 4.4 and 5.3. The temperature used in all ten studies varied from room temperature (25 °C) to 37 °C. Regarding stirring or agitation, two studies [47, 50] used EDTA solution under constant stirring for induction of artificial caries.

### Demineralizing solution followed by remineralizing solution

This different demineralizing protocol was reported in one study [40] which involved immersing the specimens in a demineralizing solution with a pH = 4.4 for 8 days, followed by a remineralizing solution with a pH = 7 for 24 h at 37 °C. Dai et al. did not clearly mention the purpose of using a remineralizing solution after the demineralizing protocol.

### Combined demineralizing protocol

In addition to using chemical or biological models for induction of artificial caries, it can be induced by using the combined protocol which involves a combination of chemical model and biological model to simulate the real conditions in the oral cavity. This is illustrated in Table 3.

**Table 3:** Combined protocol (chemical and bacterial models) used for artificial caries induction

Author(s) /Year	Chemical model				Bacterial model	
	**Demineralizing solution**	**pH**	**Time**	**Temperature**	**Bacterial species**	**Incubation time**
Steier et al*.* [38] (2022)	50 mM acetic acid, 3 mM CaCl_2_ × H_2_O, 3 mM KH_2_PO_4_ and 6 mM methyl-hydroxy-diphosphonate	5.3	12 weeks	-	*Streptococcus mutans*, *Actinomyces naeslundii* and *Streptococcus sanguis*	2 days
Schwendicke et al. [26] (2019)	50 mM acetic acid, 3 mM CaCl_2_ × H_2_O, 3 mM KH_2_PO_4_ and 6 mM methyl-hydroxy-diphosphonate	5.3	7 days	37◦C	*Lactobacillus rhamnosus*	2 days

The study by Schwendicke et al. [26]*.* used a combined protocol in one tested group for induction of artificial caries in deep dentin. The specimens were first demineralized chemically using an acetic acid solution composed of 50 mM acetic acid, 3 mM CaCl_2_ × H_2_O, 3 mM KH_2_PO_4_, and added 6 mM methyl-hydroxy-diphosphonate (MHDP) which acts as a dissolution inhibitor. The pH of acetic solution was 5.3 and the immersion time was for 7 days at 37 °C, followed by bacterial demineralization by incubation of specimens with *Lactobacillus rhamnosus* strains for 2 days at 37 °C. While Steier et al*.* [38] used the same acetic acid solution as Schwendicke et al*.* with the same pH value, but the immersion time was for 12 weeks to demineralize the specimens chemically first, then used multi-species biofilm composed of *Streptococcus mutans*, *Actinomyces naeslundii,* and *Streptococcus sanguis* for incubation period of 2 days.

### Evaluation methods for dentin mineral content

Various laboratory evaluation methods were utilized in the included studies to assess different parameters. The purpose of employing each method in these studies, along with their specifications and units of measurement, is elaborated in Tables 4 and 5 for the chemical and bacterial models, respectively. The advantages and disadvantages of each method are delineated in Table 6. Classification of laboratory evaluation methods, into quantitative and qualitative analysis, or destructive and non-destructive methods, and the principle of each method (Table 7).

**Table 4:** Different assessment methods for dentin mineral density, aim of usage, description, and unit of measurement

Author(s) /Year	Assessment method	Aim of Usage	Description	Unit of measurement
Francois et al*.* [29] (2024)	• High-energy micro-CT device	• For assessment of remineralization and to qualify various levels of demineralization/remineralization	• It was set at 90 kV and 160 μA. 3D images were acquired with 20 μm voxel size. Gray values were calibrated using a set of aqueous K_2_HPO_4_ phantoms	• MGVs
Aruna Rani et al*.* [30] (2023)	• Vickers Microhardness Indenter • HRSEM-EDX • Micro-Raman spectrometer	• To measure the surface microhardness • To assess the surface morphology and the elemental composition • To evaluate the remineralization degree	• Using a load of 20 g for 10 s at three places with a distance of 100 μm between each indent • Scanning with Apreo S operating at 20 kV under 2000 × and 5000 × magnification • A 532 nm laser source of intensity 25 mW was used with a 100 × magnification. Spectral range (500–2000 cm^−1^) was collected at a resolution of 4 cm^−1^ with a step size of 1 μm and 8 s time of exposure	• VMN - -
Cifuentes-Jiménez et al*.* [31] (2023)	• ATR-FTIR Spectroscopy • XRD • SEM EDX spectroscopy	• To measure the amount of mineral to the organic matrix and measure the crystallinity index • To measure the crystallite size of hydroxyapatite crystals of dentin mineral • To analyze the morphology and structure of dentin • To determine the elemental composition (Ca, P, Ag, I, C, O, and Na) of the dentin	• The spectral resolution was 2 cm^−1^ over 124 scan accumulations with a spectral range (600–4000 cm^−1^) • The working conditions were: Cu Kα (λ = 1.5418 Å) radiation at 50 kV and 30 mA, with a pinhole collimator of 0.5 mm in diameter • Using accelerating voltage of 20 kV and 10 mA and a magnification of 10.000–30.000x -	- • Crystallite size (nm) - -
Fernandes et al*.* [32] (2023)	-	-	-	-
Rao et al. [33] (2023)	• Vickers Microhardness Indenter • SEM	• To evaluate the mechanical properties of dentin by measuring the mineral content in dentin • To evaluate the remineralization	• Using load of 20 g for 10 s making six indents on each specimen -	• VMN -
Aldosari and Al-Sehaibany [34] (2022)	-	-	-	-
Khor et al*.* [35] (2022)	-	-	-	-
QI et al [36] (2022)	• XPS • SEM	• To investigate the surface chemical composition • To investigate the surface morphology	• Spectra were acquired at 280 eV pass energy with a step size of 1.0 eV • Scanning by using the TLD (through the lens detector) at 10 kV accelerating voltage	• Atomic percentage % -
Silva et al*.* [37] (2022)	• FTIR spectroscopy • Knoop Microhardness Indenter	• To investigate the chemical analysis of the dentin • To measure the dentin surface microhardness	• The frequency range (4000 and 650 cm^−1^), 32 scans, resolution of 4 cm^−1^. The absorbance peaks of phosphate (900—1200 cm^−1^), carbonated (870—1070 cm^−1^), and amide groups (1600—1700 cm^−1^) • Using 10-gf load for 30 s. Three indentations were performed 100, 200, and 300 μm apart	- • KHN
Steier et al*.* [38] (2022)	• Raman spectroscope • SEM	• For investigation of the chemical composition of dentin demineralized surface • To visualize the ultra-structure geometry of dentin	• The following parameters were used: 785 nm wavelength with argon ion 514.5 green laser excitation, spectral resolution of 1.6 cm^−1^, and power 500 W at 100 objectives with superior signal/noise ratio • Scanning under high vacuum at 6 kV, with 8 mm working distance, 30 m objective lens aperture, resolution of 2560 × 1920 pixels, and an acquisition time of 160 s per image	• Wavenumber (cm^−1^) -
Babaie et al*.* [39] (2021)	• Nano-indentation • SEM–EDS • Micro-CT	• To evaluate the nanomechanical properties (hardness and E-modulus) • To investigate the elemental analysis of the dentin surfaces • To determine the mineral density and average volume of the lesion	• Nanoindenter was attached to an atomic force microscope. Two rows of indents with a space of 20 μm • SEM was operated at 15 kV. Line profiles of elemental contents were recorded at 10–12 mm working distance • Scanning with resolution of 10 μm. Images were collected at 90 kV and 200 lA using 360◦ rotations, with 500 ms exposure time	- - • Gray scale values
Dai et al*.* [40] (2021)	• Micro-CT • XRD • SEM	• To measure the demineralized lesion depth and mineral loss in dentin • To assess the characteristics of the mineral crystals surface • To investigate the dentin surface morphology	• The voltage and current settings were 80 kV and 100 μA. The image pixel size was set at 7.95 μm and the X-rays were cut off by a 0.5 mm aluminium filter • XRD equipped with a CuKα lamp (λ = 1.54056 Å). The parameters were: 2θ range = 20◦ to 60◦, step size = 0.02◦ and scan speed = 0.6 s/step • SEM used under high-vacuum mode at 8000 × and 20,000 × magnification	• Lesion depth (μm) Mineral loss (g/cm^3^) - -
Abdelshafi et al. [41] (2021)	• SEM	• To investigate the dentin surface morphology to observe the irregular porous dentin surface that indicates demineralization	-	-
Cifuentes-Jimenez et al*.* [42] (2021)	• ATR-FTIR spectroscopy • XRD • SEM–EDS	• To assess potential changes in the dentin chemical composition • To study the crystalline characteristics • To observe the morphological changes in the dentin surface and tubular occlusion	• The spectral resolution was 2 cm^−1^ with 32 accumulations with a spectral range of 600–4000 cm^−1^ in absorption mode • The working conditions were: Cu K α (λ = 1.5418 Å), 50 kV, and 30 mA, with a pinhole collimator of 0.5-mm diameter • Specimens were observed at 15,000X under 20 kV SEM equipped with an EDS detector to assess	- - -
Sami et al*.* [43] (2021)	-	-	-	-
Chen et al*.* [44] (2020)	• SEM • EPMA	• To observe the surface ultra-structure of dentin collagen and the cross profile of dentin tubules • To assess the remineralization intensity and depth of lesion	• Specimens were observed under SEM at the condition of 10 kV voltage • The spatial resolution was ± 0.05 μm. The depth was detected from the occlusal surface to deeper dentin along a 300 μm long line-scan	- -
Daneshpoor and Pishevar [45] (2020)	• SEM–EDX • ATR-FTIR Spectroscopy	• To assess the surface chemistry (surface component and elemental distribution) • for analyzing the phase composition and the crystal structure	• Using an accelerating voltage of 25 kV • The spectral resolution was 4 cm^−1^ and 64 scans for each spectrum in the region of 400–4000 cm^−1^. The diameter of ATR accessory was 2 mm and the IR penetration power was about 2 μm	• Weight% and atomic % -
Sadoon et al*.* [46] (2020)	SEM–EDX Surface Microhardness Test Optical microscope	• To evaluate the mineral content in dentin • To measure the surface hardness • To examine areas of sound, demineralized, and remineralized dentin	- • Using a load of 25 g and time of 10 s • It was connected to a digital camera	• Weight percentage % • Kg.mm^−2^ -
Scholz et al*.* [47] (2020)	Micro-CT	• To determine the volume of demineralized dentin	• The scanning parameters: duration = 170 min, Images = 3,000, Averaging = 15, tube voltage = 80 kV, Skip = 2, beam current = 500 μA, voxel size = 35 μm	• Mm^3^
Wu et al*.* [48] (2020)	• AFM • Surface Microhardness Test • CLSM	• To investigate surface morphology changes • To measure the surface hardness • To evaluate the remineralization depth of dentin carious lesion	• Observation was done in 4 different sites. A field of view at 50 μm × 50 μm scan size, 1.5 Hz scan rate, and a resolution of 512 by 512 pixels • Using loads of 0.98 N and time for 15 s and measured at three widely positioned locations • Standard settings for contrast, brightness, and laser power were used	- • VMN • Lesion depth (μm)
Zhao et al*.* [49] (2019)	-	-	-	-
Schwendicke et al. [26] (2019)	• TMR • Vickers Microhardness Intender • Fluorescence Optical • Microscope and CLSM • TEM	• To estimate the integrated mineral loss and gain in the specimens • To measure the surface hardness • To assess the interfacial characteristics of the demineralized/remineralized dentin interfaces • Used for evaluating the overall effect of remineralization	• It was operating at 20 kV and 10 mA with a vertical tube and a 280 mm radiation-to-film distance • Using a load of 1 N (100 g) for 15 s • Optical microscope was equipped with an LED light, a filter-pass (490–520 nm), and a 20 × NA 0.7 oil-immersion lens. CLSM was equipped with a 63 ×/1.4 NA oil-immersion lens and a 514 nm argon/helium ion laser • Examination was performed using at 110 kV	• vol% × μm • VMN - -
Saxena et al*.* [50] (2019)	• XRD • SEM–EDS • TGA • TEM	• To determine crystal structure To determine the elemental composition of the surface • To measure the mineral weight • To access different depths below the surface of the specimens	• Scanning performed from 10° to 60° (2ϴ) with a step size of 0.01° and time of 10 s/step using Cu Kα x-rays (*λ* = 1.54 Å) - • It was performed under nitrogen up to 600 °C at a heating rate of 20 °C/min using a TA Instruments Q5000 • Sections of specimens were prepared via FIB with a Strata DB235 Dual-Beam instrument	- - - • Weight %

*Abbreviations:* *Micro-CT* Micro-computed tomography, *MGVs* Mean gray values, *VMN* Vickers microhardness number, *HRSEM-EDX* High resolution scanning electron microscope—energy dispersive x-ray, *ATR-FTIR* Attenuated total reflectance- fourier transform infrared, *XRD*: X-ray diffraction, *SEM* Scanning electron microscope, *EDX* Energy dispersive x-ray, *XPS* X-ray photoemission spectroscopy, *FTIR* Fourier transform infrared spectroscopy, *KHN* Knoop hardness number, *SEM–EDS* Scanning electron microscope-energy dispersive x-ray spectroscopy, *EPMA* Electron probe micro analyzer, *AFM* Atomic force microscope, *CLSM* Confocal laser scanning microscopy, *TMR* Transverse microradiography, *TEM* Transmission electron microscopy, *TGA* Thermo-gravimetric analysis, *FIB* Focused ion beam

**Table 5:** Assessment methods for bacterial model in combined protocol, the aim of usage, description, and unit of measurement

Author(s) /Year	Assessment method	Aim of usage	Description	Unit of measurement
Steier et al*.* [38] (2022)	• CFU • CLSM • FISH	• To measure the bacterial counts which used to examine the growth kinetics of biofilms • To analyze the invasion of bacterial biofilms into the dentinal tubules • For visualization of bacteria on enamel and dentin	• Biofilms were grown for 7 days on dentin discs. Dentin biofilms were collected in 1 mL of sterile BHI broth (pH 7.4) and cultured for 24 h at 37 ◦C. 100 L of broth were centrifuged five times in 100 L of PBS inside Eppendorf tubes. Each diluted specimen was plated on selective BHI agar plates in 5 µl and incubated for 24 h • Images were captured at various magnification and an electronic zoom of 6.3 times. Vertical sectioning at 0.97-m intervals through the biofilm produced a Z-series of optical sections -	• Number of microbial colony-forming units per milliliter (CFU/mL) - -

*Abbreviations*: *CFU* Colony-forming units, *BHI* Brain heart infusion, *PBS* Phosphate-buffered saline, *FISH* Fluorescence in situ hybridization

**Table 6:** Advantages and disadvantages of each evaluation method

No	Evaluation methods	Advantages	Disadvantages
1	Micro-CT	• The possibility of multiple scanning and image processing • Specimen preparation is not an issue as it is a non-destructive method • Reproducible, accurate technique and precise measurements	• Long scanning and processing time • High cost • Necessity of computer expertise • The image file sizes are too large • High radiation dose
2	Microhardness Nanoindentation	• Simple technique • Relatively low cost • Accurate measurements • Diamond tip does not deform over time • Long research experience	• The surface to be investigated must be very well polished and flat for accurate measurements • Deposition of fluoride on the surface may lead to inaccurate measurements
3	SEM	• High-resolution 3D imaging • High magnification power • Easy to operate with proper training	• Destructive approach, require specimen preparation as coating and desiccation • High cost
4	SEM/EDX	• Powerful analytical technique • High spatial resolution	• Require specimen preparation in a high vacuum • Expensive • Not differentiate between atomic and non-atomic species
5	Raman Micro-Raman	• Non-invasiveness, no specimen preparation • High biochemical specificity • Low water sensitivity • Fast, need a short duration time	• High cost • Shallow penetration depth of the light beam • Fluorescence affects the quality of Raman spectra
6	XRD	• Non-destructive nature • High sensitivity and reliability • Easy specimen preparation • Rapid, powerful, and effective technique	• Specimen must be single phase and homogeneous • It characterizes only crystalline materials • High cost • Harmful radiation
7	ATR-FTIR	• Fast, no time consuming • Non-invasive and relatively simple measuring technique • Inexpensive • Easy to be operated • Precise measurements	• Equipped only with a single beam, whereas dispersive instruments generally have a double beam • Limited penetration depth (1–10 μm)
8	EPMA	• Identify the concentration of elements within a small area of the specimen • Simple and accurate method	• Destructive specimen preparation through desiccation and coating • The accuracy of EPMA is dependent on the homogeneity of the specimen
9	CLSM	• Direct and non-invasive approach • Optical sectioning of the specimen with 3D reconstruction • Fast recording of surface topography • Reproducible measurements • No specific specimen preparation	• High cost • Low magnification (max. × 1000) resolution limited to the optical diffraction limit
10	TMR	• Most practical and direct technique • Most informative approach • High sensitivity in thinner sections	• Destructive technique • Not give morphological data • Time consuming (long exposure time) • Difficult to be performed on fragile lesion surfaces
11	TEM	• High-quality image of internal structure • High resolution and magnification	• Destructive specimen preparation (ultra-thin sections of the specimen are required) • Time consuming • Require experienced experimentalist
12	TGA	• Convenient and time-saving technique • High accuracy of balance • Continuous recording of weight loss as a function of temperature ensures equal weightage Relatively low cost	• Limited to specimens that undergo weight change • Very sensitive to any change • Require high control over temperature • Destructive as a result of using very high temperature
13	XPS	Effective approach Surface-sensitive analysis Non-destructive analysis, no or little specimen preparation	• It is associated with a 10% relative error in repeated analyses • It is expensive due to its highly sensitivity
14	AFM	• Able to study wet surfaces so no artifacts are caused due to dehydration • Provide information about dentin roughness • No surface preparation	• Not suitable for the estimation of properties of larger areas on a surface • High cost • Time-consuming Need specialist
15	Optical microscope	• Simple approach • Relatively low cost • Ease of microscope handling	• Low resolution

*Abbreviations:* *Micro-CT* Micro-computed tomography, *SEM* Scanning electron microscope, *SEM/EDX* Scanning electron microscope-energy dispersive x-ray, *XRD* X-ray diffraction, *ATR-FTIR* Attenuated total reflectance- fourier transform infrared, *EPMA* Electron probe micro analyzer, *CLSM* Confocal laser scanning microscopy, *TMR* Transverse microradiography, *TEM* Transmission electron microscopy, *TGA* Thermo-gravimetric analysis, *XPS* X-ray photoemission spectroscopy, *AFM* Atomic force microscope

**Table 7:** Qualitative and quantitative analysis, destructive and non-destructive laboratory evaluation methods of dentin mineral density with the principle of each method

No	Evaluation methods	Qualitative/Quantitative analysis	Destructive/Non-destructive	Methods’ principals
1	Micro-CT	Quantitative	Non-destructive	• It used micro-focal spot X-ray sources and high-resolution detectors, allowing for projections rotated through multiple viewing directions to produce 3D reconstructed images of specimens. The images represent spatial distribution maps of linear attenuation coefficients determined by the energy of the X-ray source and the atomic composition of the material specimen
2	Microhardness Nanoindentation	Quantitative	Non-destructive	• Diamond tip is applied to the specimen under a certain load and for a certain period, measuring the imprint of the probe, and calculating a hardness value The nanoindentation test follows the same principle as the microhardness test but on a smaller scale
3	SEM	Qualitative	Destructive	• It generates images by recording the BSE. The elastically scattered electrons are returned to the primary material from the surface so contrast between regions of different compositions is achieved. In this way, accurate information about the morphology of analyzed biomaterials could be obtained
4	SEM/EDX	Qualitative/semi-quantitative	Destructive	• The specimen is bombarded by a high-voltage electron beam. The interaction between them causes an emission of radiation in the X-ray range, which is characteristic of an element. This allows high-speed qualitative elemental analysis. Quantitation can also be made according to the intensity of the energy emitted by the specimen
5	Raman Micro-Raman	Quantitative	Non-destructive	• It is used to measure the vibration frequency of scattered light from specific component molecules when the incident light hits them. Each organic molecule has its own specific vibration frequency, which is visualized in the form of peaks
6	XRD	Quantitative	Non-destructive	• When X-ray falls over a specimen, it diffracts in a pattern characteristic to its structure. Every material has its unique diffraction patterns so materials and compounds can be identified by using a database of diffraction patterns
7	ATR-FTIR	Quantitative	Non-destructive	• FTIR spectrometer is coupled with ATR accessory. In ATR sampling, the IR light travels through a crystal, is totally internally reflected at the crystal-specimen interface, and the reflected light travels to the FTIR detector. During the internal reflection, a part of the IR light travels into the specimen, where it can be absorbed
8	EPMA	Qualitative/quantitative	Destructive	• It irradiates a focused electron beam on a specimen and collects the X-ray photons emitted at various elemental chemical species by the electron beam. The chemical composition of the specimen is identified by the wavelength spectrum of the emitted X-ray photons
9	CLSM	Qualitative/semi-quantitative	Non-destructive	• It is fluorescence microscope that records light emission under a focused scanning laser beam at a fixed focal plane. The specimens absorb the light and re-emit it with longer wavelengths. The emissions are recorded by a detector and then converted to an image. Specimens must be labeled with fluorescent marker such as rhodamine dye
10	TMR	Quantitative	Destructive	• It depends on transversally sectioning the specimens into very thin slices. Monochromatic x-rays contact each slice, together with a calibration step-wedge. The x-ray adsorption was reflected directly in the optical density of the film to generate the images. By using micro densitometry, the mineral content in vol.% could be calculated
11	TEM	Qualitative	Destructive	• When the electron beam is transmitted through the specimen, some electrons are absorbed or deflected. The areas where more electrons pass through, create bright spots on the screen below, and the areas where fewer electrons come through create darker spots. This, in turn, creates a magnified, shadow-like, black and white image of the specimen
12	TGA	Quantitative	Destructive	• It determines the amount and the rate of weight change of a substance with respect to temperature or time in controlled programmed conditions. The mass change profile (loss or gain) is recorded as the specimen is subjected to a controlled heating or cooling
13	XPS	Quantitative	Non-destructive	• Photoelectrons are emitted from the specimen in response to electromagnetic radiation. An electron energy analyzer is used to measure the energy of the emitted photoelectrons. From the binding energy and intensity of a photoelectron peak, the elemental identity, chemical state, and quantity of a detected element can be determined
14	AFM	Qualitative	Non-destructive	• It uses an ultra-small probe tip at the end of a cantilever. It scans the surface of specimens with a probe and this interaction is used to measure fine surface shapes or properties. The measurement of AFM is made in three dimensions, the horizontal X–Y plane, and the vertical Z dimension
15	Optical microscope	Qualitative	Non-destructive	• It is used to closely view a specimen through the magnification of a lens with visible light. This is the traditional form of microscopy

*Abbreviations:* *Micro-CT* Micro-computed tomography, *SEM* Scanning electron microscope, *BSE* Backscattered electrons, *SEM/EDX* Scanning electron microscope-energy dispersive x-ray, *XRD* X-ray diffraction, *ATR-FTIR* Attenuated total reflectance- fourier transform infrared, *EPMA* Electron probe micro analyzer, *CLSM* Confocal laser scanning microscopy, *TMR* Transverse microradiography, *TEM* Transmission electron microscopy, *TGA* Thermo-gravimetric analysis, *XPS* X-ray photoemission spectroscopy, *AFM* Atomic force microscope

### Techniques used in included studies

Seven studies [30, 31, 39, 42, 45, 46, 50] used scanning electron microscope-energy dispersive X-ray spectroscopy (SEM–EDX) (30%). Six studies employed surface microhardness test (26%), five of them [26, 30, 33, 46, 48] utilized Vickers microhardness indenter (22%), while one study [37] used Knoop microhardness indenter (4%), and another study [39] employed nano-indentation (4%). Micro-CT devices were used in four studies [29, 39, 40, 47] (17%), while micro-Raman devices were used in two studies [30, 38] (9%). X-ray diffraction (XRD) was used in four studies [31, 40, 42, 50] (17%), and attenuated total reflectance-Fourier transform infrared (ATR-FTIR) devices were used in four studies as well [31, 37, 42, 45] (17%). Transmission electron microscopy (TEM) was reported in two studies [26, 50] (9%). Scanning electron microscopy (SEM) was used in six studies [33, 36, 38, 40, 41, 44] (26%), confocal laser scanning microscopy (CLSM) was used in two studies [26, 48] (9%), atomic force microscope (AFM) was used in one study [48] (4%), X-ray photoemission spectroscopy (XPS) was used in one study [36] (4%), and transverse microradiography (TMR) was used in one study [26] (4%). Thermo-gravimetric analysis (TGA) was reported in one study [50] (4%), and electron probe micro analyzer (EPMA) was used in one study [44] (4%). Additionally, three studies [26, 31, 46] reported the use of an optical microscope (13%). Fifteen studies of the included studies [26, 30, 31, 33, 36–40, 42, 44–46, 48, 50] used a combination of multiple evaluation methods (65%). Three studies [29, 41, 47] reported using only one analytical approach to evaluate mineral content (13%), whereas five studies [32, 34, 35, 43, 49] did not employ any evaluation method specifically related to dentin mineral assessment (22%).

## Evaluation parameters

### Dentin mineral density

Mineral profile changes of dentin involving mineral loss or gain, can be evaluated by various methods such as micro-CT, TMR, micro-Raman, and TGA. Micro-CT is considered the gold standard 3D non-destructive imaging approach. It provides reliable, reproducible, and accurate information about the distribution patterns of mineral density, allowing for the determination of a standard mineral density value [51]. Additionally, micro-CT enables quantitative measurement of mineral loss after demineralization and mineral regain after remineralization [52]. It has been utilized in four studies to investigate different parameters [29, 39, 40, 47], including assessment of remineralization/demineralization levels and evaluation of volumetric demineralized lesions. The study by Dai et al. [40] used a micro-CT device to evaluate depth of lesion, as well as amount of mineral loss.

TMR is considered as the gold standard 2D technique for determination of mineral density and lesion depth [21]. It is a reliable and highly sensitive destructive approach for quantifying the amount of mineral loss or gain from dental substrate [26, 53], as well as quantifying the thickness and degree of the surface layer mineralization [54]. The mineral concentration profile is used to calculate lesion depth and integrated mineral loss or gain after remineralization [55]. The data obtained from TMR analysis are expressed as the volume percentage (vol%) [56].

Micro-Raman was used in one study [30] to assess different levels of mineral changes and evaluate the degree of remineralization. This non-destructive and non-invasive optical method is considered as a reliable technique for analyzing molecular composition and identifying deposited minerals. Furthermore, it provides accurate quantitative information, particularly regarding phosphate, carbonate, and amide groups, which are relevant vibrational peaks. The study by Steier et al*.* [38] used Raman spectroscopy to characterize chemical composition of demineralized dentin surfaces.

Mineral weight was evaluated quantitatively in one study [50] using TGA. It is a destructive method used to measure mineral amount and the rate of weight change with respect to temperature or time under controlled programmed conditions [57]. By comparing the mass loss between different temperature ranges, it is possible to precisely quantify the amount of each phase present [58]. Saxena et al*.* [50] utilized TGA to measure mineral weight of specimens through firing process at 600 °C, which resulted in complete combustion of collagen, leaving only mineral residues.

Elemental analysis and chemical composition were evaluated in ten studies [30, 31, 36, 38, 39, 42, 44–46, 50] using different evaluation methods such as EDX, EPMA, ATR-FTIR, XPS, and Raman spectroscopy. EDX is considered as a gold standard analytical destructive approach and the most commonly used device for investigating elemental distribution and dentin surface chemistry, as reported in six studies [30, 31, 39, 45, 46, 50]. It is considered a semi-quantitative method that provides approximate measurements rather than an exact measurement [59, 60]. When combined with SEM, EDX can determine Ca and P weight percentages, allowing calculation of Ca/P ratio. It can detect the deposition of active agents from therapeutic treatments on the tooth surface [53].

There is a special type of electron microscopy (EPMA) used for elemental analysis of hard tooth structure. It is considered a destructive method that provides qualitative and quantitative information on the chemical composition, with a sensitivity level in parts per million (ppm) [61]. It offers accurate information about the distribution of elemental compositions, particularly calcium and phosphate levels in weight percentage (wt.%) [56]. In addition to assessing the remineralization intensity, EPMA can evaluate the depth of demineralized lesions, as mentioned in one included study [44].

Three studies [31, 37, 42] utilized ATR-FTIR to assess potential changes in the chemical composition of dentin after applying the treatment agents. FTIR spectroscopy is used to determine the characteristic vibrations of atomic groups of the studied compound. It shows the chemical bonds present in a specimen, allowing for the identification of its chemical nature. The ATR technique enables the penetration of the light beam into a specimen depth of about 0.5–3 μm [23, 55]. ATR-FTIR is considered a quantitative, reliable, and non-destructive technique for identifying the deposited mineral. It is a highly sensitive tool for studying the changes in surface composition at the molecular level and enables easy characterization with little or no specimen preparation [62].

The study by QI et al*.* [36] used XPS to investigate the surface chemical analysis of dentin. It is considered a non-destructive quantitative method used to provide elemental information about the surface. It is suitable for chemical state identification of surface species [23, 63]. Qualitatively, SEM and TEM were used in three studies [26, 33, 50] to assess the overall remineralization effect by observing the precipitation of crystals at different depths.

#### Depth of lesion

Evaluation of lesion depth or remineralization depth was reported in three studies [40, 44, 48] using three different evaluation methods, including micro-CT, EPMA, and CLSM. The study by Dai et al. [40] scanned the enamel and dentin lesions using micro-CT and the depths of lesions were measured by reconstruction software, while Chen et al*.* [44] used EPMA to measure the depth and intensities of chemical elements Ca and P from the occlusal surface to deeper dentin along a 300-long line scan. Through the EPMA line scans, it detects the beginning and end of demineralized artificial caries dentin lesions showing a demineralization depth of 150 ± 50 μm. They concluded that dentin was partially demineralized by a pH-cycling protocol to a depth of 150 ± 50 μm starting from the uncovered occlusal surface. Wu et al*.* [48] quantitatively analyzed the remineralization depths by using CLSM with an image-analysis system. Presence of a fluorescent band can indicate the depth of lesion or remineralization depth. The observation of CLSM showed a red fluorescent band representing the caries lesion, while the decrease in fluorescence on the superficial layer of the lesion indicated remineralization.

In the included studies, various authors discussed different levels of lesion depth which were achieved through using different demineralization protocols. For instance, Fernandes et al*.* [32] indicated that utilizing acetic acid as a demineralizing solution with a pH of 4.5 for 2 days resulted in a demineralized dentin surface with a depth of approximately 100 μm. On the other hand, Babaie et al*.* [39] employed acetic acid solutions with varying pH values (5 and 4.5) and immersion times (66 h and 168 h) to produce shallow (~ 140 μm) and deep lesions (~ 700 μm), respectively. Rao et al. [33] reported that using a pH-cycling model for 14 days led to a demineralization depth exceeding 100 μm, while Chen et al*.* [44] stated that dentin was partially demineralized to a depth of 150 ± 50 μm through a 14-day pH-cycling model. Similarly, Francois et al*.* [29] utilized a pH-cycling model for 14 days to demineralize dentin specimens to an average depth of 158.3 ± 30.9 μm. In contrast, Sadoon et al*.* [46] stated that the use of a pH-cycling model resulted in dentin lesions of 40 μm depth, shallower than natural caries lesions.

#### Crystalline structure

Only crystalline materials can be characterized by XRD technique [23]. It is considered as a non-destructive quantitative technique used to analyze physical properties such as phase composition and crystalline structure. Since every material has its unique diffraction patterns, materials can be identified by using a database of diffraction patterns [64]. The diffraction pattern of the crystalline materials is well-defined, narrow, sharp and exhibits significant peaks, while amorphous materials do not exhibit clear peaks; rather, the pattern shows noise signals and smeared peaks [64]. Two studies [40, 50] evaluated the crystalline structure and the characteristics of surface mineral crystals using XRD, and one study [45] utilized ATR-FTIR for the same purpose. Additionally, two studies [31, 42] employed both XRD and ATR-FTIR for evaluating the crystallite size (i.e. estimation of the size of the coherently scattering domains of crystals) and crystallinity index (i.e. the ratio of the crystalline peaks to the crystalline + amorphous peaks), respectively.

#### Surfaces microhardness

Seven studies [26, 30, 33, 37, 39, 46, 48] evaluated surface microhardness and mechanical properties of dentin surfaces. The microhardness test and nanoindentation test were considered as non-destructive laboratory tests that provided accurate and reliable indirect information about mineral changes in sound, demineralized, and remineralized dentinal surfaces [8]. In demineralized dentin, a marked reduction in mechanical properties was associated with a decrease in its mineral content. Microhardness test was performed by applying a specific load on specimen for a specific period of time, measuring the imprint of the probe, and calculating a hardness value [55]. Two types of microhardness indenters were used according to the shape of the probe (Vickers and Knoop); Vickers microhardness indenter (tetra-pyramidal in shape) was used in five studies [26, 30, 33, 46, 48], while Knoop microhardness indenter (rhomboidal in shape) was used in one study [37].

One of the included studies [39] employed a nanoindentation test, which follows the same principle as the microhardness test but on a smaller scale [53]. The nanoindentation test was used to indirectly determine the depth of the lesion by measuring the nanomechanical properties of demineralized and remineralized dentin surfaces [65]. It was also used to measure Young’s modulus (elastic deformation) as mentioned in one study [39]. Nanoindentation of dentin is often combined with AFM for better visual control and to identify the shape and size of the resulting indentations, since nano-indenters are too small to be placed on peri- or inter-tubular dentin or even inside a dentinal tubule [39, 53].

#### Surface morphology

The most popular imaging technique used to observe the surface texture morphology and ultrastructure characteristics of dentin surfaces was performed using SEM [66], as reported in eight studies [30, 31, 36, 38, 40–42, 44]. It is considered a destructive, qualitative, and ideal descriptive method to evaluate the interface between the tooth structure and restoration [67]. It provides sufficient information related to demineralization and mineral precipitation. The SEM micrographs yield a characteristic three-dimensional representation that is useful for understanding the surface morphology of specimen [68, 69]. SEM investigations have been used to reveal the effects of superficially deposited precipitates resulting from mineral dissolution by various agents [65].

Confocal Laser Scanning Microscopy (CLSM) is one of the most important devices relays on fluorescent imaging field [70]. The areas of fluorescence and total fluorescence of carious lesions measured by CLSM have shown mineral loss related to pore space infiltrated by fluorescent dye [71]. Rhodamine is the most commonly used dye as its absorption peak (511nm) is compatible with the wavelength of the majority of the excitation lights used in confocal laser microscopy [67]. CLSM provides high-resolution and high-contrast three-dimensional optical images compared to the light conventional microscope, with depth selectivity from specimens [67, 72]. It is considered a non-destructive qualitative/semi-quantitative method capable of quantifying and visualizing caries lesions. It was used in one study to qualitatively investigate the interfacial characteristics of dentin surface [26], additionally, it was used to quantify the remineralization depth of dentin carious lesions combined with AFM to visualize the surface morphology as mentioned in another study [48]. The use of fluorescence microscopy techniques has been reported for bacterial visualization in enamel and dentin. This was performed by Steier et al*.* [38], who used CLSM in conjunction with fluorescence in situ hybridization (FISH) to visualize the invasion of bacterial biofilms into the dentinal tubules.

The TEM technique is described as a predecessor of SEM [67], and one of the most commonly used techniques to analyze the interfacial conditions. It is considered a destructive qualitative approach used to characterize the interfacial ultra-structure changes in dentin [73]. Two studies [26, 50] reported using TEM to assess the overall remineralization effect at different depths below the surface by observing the precipitation of crystals. An optical microscope is a useful tool for examining general tooth anatomy and the basic features [74]. It was utilized in three studies [26, 31, 46] to qualitatively examine the areas of sound, demineralized, and remineralized dentin surfaces using teeth ground sections.

AFM has been applied in investigations of the ultra-morphology of superficial and deep dentin and its mechanical properties [75]. It is considered a non-destructive qualitative approach based on mapping an atomic-force field on the surface of the examined specimen [67]. It provides a real topographical three-dimensional image of the specimen surface and information about dentin roughness [72, 75]. It is often used to identify the sites of nano-indentations in nanoindentation tests, as mentioned in one of the included studies [39]. The study by Wu et al. [48] employed surface roughness test using AFM for identification of the morphological changes of the surface.

#### Assessment of risk of bias

**Fig. 3:**
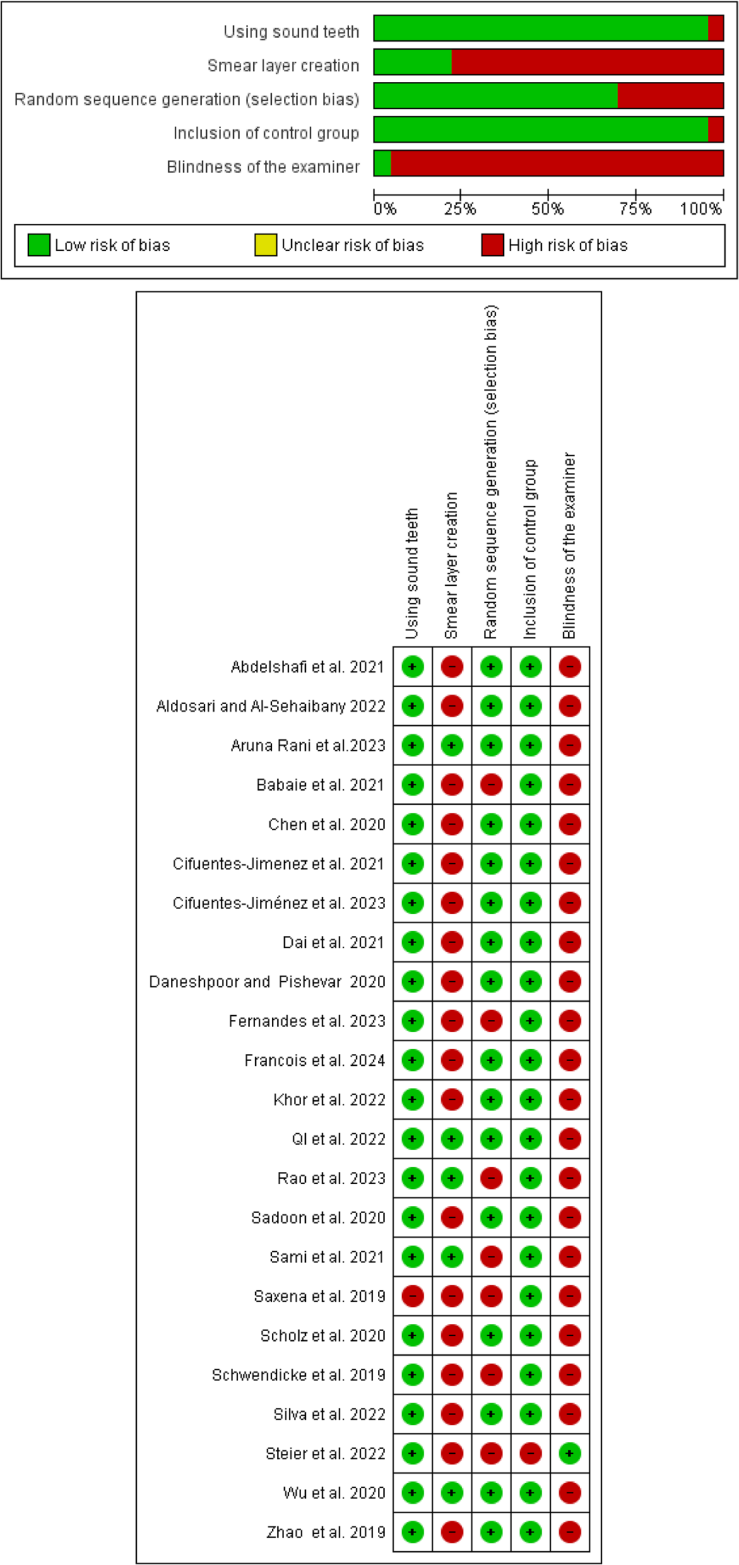
Risk of bias graph and summary

Based on the parameters outlined in the analysis, three studies [30, 36, 48] demonstrated a low risk of bias (13%), while fifteen studies [29, 31, 33–35, 37, 40–47, 49] exhibited a medium risk of bias (65%). The remaining five studies [26, 32, 38, 39, 50] indicated a high risk of bias (22%). The most frequently utilized parameter in the included studies is the use of sound/healthy teeth, whereas the least utilized parameter is the blinding of the examiner. The risk of bias graph and summary are illustrated in Fig. 3.

## Discussion

Evidence-based dentistry is an approach that involves systematically collecting and analyzing scientific evidence to address specific clinical questions [76]. Since individual studies may not provide conclusive answers, systematic reviews are utilized to compare results from multiple studies and determine the most reliable clinical evidence [28].

Laboratory models are the most commonly used approach for studying the demineralization/remineralization process in cariology research [17]. Inducing artificial caries lesions through laboratory models, whether in dentin or enamel, has been developed to understand various aspects of caries lesions and their etiology [12]. Establishing a consistently formed and reproducible laboratory lesion is crucial for comparing treatments and evaluating outcomes. It has been suggested that the mechanical properties of caries-affected dentin, which are related to mineral content, significantly influence bond strength to the substrate [20]. Therefore, remineralization of demineralized caries-affected dentin could enhance bond strength [33], which is vital for biomimetic and preventive dentistry, which aims to conserve dental tissues and reconstruct the remaining tooth structure [77].

This systematic review primarily focuses on discussing chemical models used on human dentin for inducing artificial caries due to their simplicity, affordability, reproducibility, and high scientific acceptance. In contrast, the lack of consistency and reproducibility in bacterial models can be a drawback when compared to chemical models [17]. Inclusion of a limited number of studies involving the use of a combined protocol is due to its rarity within the research inclusion criteria. Additionally, it is a more complex and time-consuming procedure compared to other protocols. Furthermore, the review examines various laboratory evaluation methods for assessing different parameters, such as dentin mineral density and demineralization depth in artificial caries dentinal lesions.

The authors have restricted the inclusion of studies in this systematic review to the last five years due to the existence of another systematic review that covers studies published from 2006 to 2021 [55]. This restriction enables discussion of the latest results from the included studies. Following the inclusion/exclusion criteria, the authors excluded demineralization protocols that utilized strong acids such as hydrochloric acid, phosphate acid, and citric acid since they are mainly employed to induce acid erosion [17]. Bovine teeth were also excluded due to differing chemical characteristics and structure compared to human teeth [78]. Additionally, assessment methods focusing on evaluating the bonding interface or collagen, as well as evaluation methods for assessing the treatment material itself, were excluded as they are not pertinent to the research questions, which particularly aim to determine the appropriate evaluation procedure for assessing dentin mineral density and the depth of artificial dentinal lesions.

According to the outcome of this review, pH-cycling model emerged as the most commonly used method (48%) for inducing dentin demineralization and evaluating the response of treatment materials to caries. The most frequently used demineralizing solution to demineralize dentin at a lesion depth of 100–150 μm is acetic acid with pH values ranging from 3.5 to 4.8 [29, 33, 44]. Concerning immersion time, specimens were immersed for a shorter duration in demineralizing solution with a low pH, which was correlated to the period of exposure to bacterial acids. In contrast, the immersion time in the remineralizing solution is longer with a constant pH, aligning with intervals between acid exposures dwell intervals [19, 79]. It was observed that pH-cycling model generates shallower caries lesions compared to natural carious lesions [11]. This was explained by absence of bacterial involvement leading to incomplete chemical simulation of oral cavity conditions.

Simple demineralization model was identified as the second most commonly used method (44%) for inducing artificial dentin caries following the pH-cycling model. This is attributed to its simplicity, cost-effectiveness, time-saving nature, and fewer operational steps required. However, it lacks a microbiological component [17]. The model relies on the use of basic demineralizing agents such as acetic and lactic acids. These acids are simulating the metabolic by-products from oral biofilms which exhibiting capability of dissolving minerals and accelerating resin hydrolysis [80]. Some of the included studies utilized ethylenediaminetetraacetic (EDTA) acidic gel to create carious lesions [45, 47, 50]. The rationale behind using gel consistency was due to its slower ion diffusion rate compared to traditional demineralizing solutions [17]. It was reported that the use of highly concentrated EDTA chelating agents induced deep lesions with steep mineral gradients and nearly complete mineral loss within the lesion body [81]. The lesion depth and impact of mechanical stirring are strongly correlated with increased agitation enhancing mobility and ion exchange and consequently accelerating tooth demineralization [19]. Dai et al. [40] introduced a different demineralization protocol and they did not clearly explain the purpose of using the remineralizing solution for one day after the demineralization process. Accordingly, it was not categorized as pH-cycling model due to the absence of alternating demineralization and remineralization cycles.

The results of the included studies showed that using multiple evaluation methods in each study exhibited confirmatory results between different treatment groups and multiple assessment devices used. There were no conflicts in outcomes among the included studies regarding different assessment devices utilized. The high demand for using a non-destructive method promotes the use of micro-CT for evaluation of mineral density instead of TMR [54]. This was attributed to the destructive nature of TMR which involves ultra-thin specimen preparation for 50–200 μm in order to precisely detect mineral changes and lesion depth [53]. However, microcracks that may occur during the sectioning process can impact the evaluation's validity [71, 82]. It is necessary to mention that TMR requires less scanning time in comparison with micro-CT which minimizes the possibility of dentin shrinkage [54].

Assessment of lesion depth and acquiring spatial information from artificial dentin caries lesions is essential in the current context to evaluate the effectiveness of chemical interventions and assess the efficacy of remineralizing agents [83]. Based on the outcome of this study, the depth of demineralization/remineralization can be quantitatively or qualitatively assessed using various methods such as micro-CT, TMR, EPMA, and CLSM. The study by Soares dos Santos et al. [54] argued that micro-CT may not be appropriate for estimating lesion depth, it can measure dentin mineral loss regardless of the degree of demineralization, whereas TMR is better suited for determining lesion depth. In contrast, Dai et al*.* [40] utilized micro-CT to measure mineral loss and the depth of demineralized lesions in both dentin and enamel. Zhi and Itthagarun [84] concluded that the lesion depth obtained from micro-CT differs from that of TMR, possibly due to the quality of micro-CT data influenced by contrast resolution and spatial resolution. The outcome of TMR analysis conducted by Soares dos Santos et al*.* [54] revealed that integrated mineral loss and lesion depth were calculated based on grey values, assuming that sound dentin contains 50 vol% mineral and the lesion ends when dentin's mineral content reaches 95%, equivalent to 47.5 vol% [21]. In micro-CT analysis, values of lesion depth were determined by defining the lesion bottom where mineral density is stabilized (matching the mineral density of sound dentin, which is 50 vol%) [54].

Both EDX and EPMA methods are utilized for elemental analysis and are regarded as destructive methods as they necessitate specimen preparation involving sectioning and desiccation [61]. It provides semi-quantitative analysis by utilizing larger electron-focusing coils and detectors positioned at a lower angle, resulting in a longer X-ray path through the specimen [85]. In contrast, EPMA employs a Wavelength-Dispersive X-ray detector instead of an EDX detector. The study by Ngo et al*.* [56] concluded that EPMA provides a simple and accurate approach to determine the depths of artificial caries lesions in dentin.

In recent years, some authors have stated that specific areas of teeth, such as dentinal tubules and the enamel layer with a higher calcium concentration, are better visualized through vibrational spectroscopic elemental maps than other optical images like radiographs or electron microscopy, which offer low-resolution molecular structural information [25]. Vibrational spectroscopy techniques (Raman and FTIR) are utilized to identify changes in chemical composition by acquiring spatially resolved spectra with micron-scale resolution [25]. ATR-FTIR spectroscopy has proven to be a highly sensitive tool for studying molecular-level changes in surface composition. Additionally, it allows for repeated analyses of the same surface location, ensuring high comparability between spectra before and after specimen treatment [62]. Raman spectroscopy, in particular, is highly specific and sensitive in the biomedical field, with the spectral changes it detects being unique to specimen analyzed and often referred to as"fingerprints"[86].

The mineral phase of enamel and dentin consists of a calcium phosphate phase derived from hydroxyapatite [55]. XRD is employed to identify this phase, characterize the surface mineral crystals, and determine crystallinity by comparing the integrated intensity of the background pattern to that of the sharp peaks [64], and measure the crystalline size of hydroxyapatite crystals. Combining XRD with vibrational spectroscopy techniques such as FTIR or Raman provides a comprehensive chemical characterization of the studied area [55].

According to the results of this review, the surface microhardness test has been identified as the most common, simplest, and cost-effective method that offers indirect information about the mineral content in tooth structure (26%) [8, 53, 87]. The effectiveness of a demineralizing protocol used to simulate caries-affected dentin can be confirmed by observing a decrease in the hardness values of demineralized dentin compared to sound dentin [11]. Conversely, an increase in microhardness values in remineralized specimens after treatment indicates an increase in mineral density and the effectiveness of the remineralization process [11]. In comparison with TMR, TMR is particularly useful for dentin due to its composition, which is rich in organic content and water [54]. The demineralization process of dentin involves the chemical dissolution of inorganic material and degradation of the organic matrix. Consequently, the high organic content of dentin may impact the measurement of its mechanical properties [21]. Therefore, using the microhardness test as an alternative to TMR may lack accuracy. However, combining these two methods not only provides information on the mechanical properties of tooth structure but also enables the estimation of mineral content (loss or gain) [88].

The outcomes of this review revealed that the most commonly utilized approach for morphological analysis is SEM (26%), often combined with EDX for elemental analysis. This pairing enables the calculation of the Ca/P ratio and provides information about the chemical composition of specimens based on the characteristic X-rays emitted under electron bombardment [30, 31, 39, 42, 45, 46, 50]. However, due to its destructive nature involving high vacuum and desiccation, leading to drying and shrinkage artifacts [53], alternative microscopic techniques such as CLSM have been employed. CLSM captures more details than SEM due to its non-invasive nature, constant maintenance of specimens under humid conditions, and its ability to perform optical sectioning. Nevertheless, CLSM exhibits shadowing effects in deeper regions, relatively poor resolution [89], and significantly lower maximal magnification compared to SEM, TEM, and AFM.

When comparing TEM and SEM, SEM generates images using electrons ejected from the specimen, scanning the surface images, while TEM captures images of the internal composition. Consequently, TEM specimens must be thinly sliced for electrons to pass through, making specimen preparation more challenging than that for SEM [90]. Hence, TEM is considered a destructive method, requiring mechanical polishing or focused ion beam techniques to achieve the required thickness (100 nm or less) [73]. The specimen's thickness determines the resolution and image quality [67]. The study by Schwendicke et al. [26]*.* used TEM in conjunction with CLSM to observe the remineralization effects of treatment materials, revealing significant mineral precipitation in treated dentin in the form of irregularly shaped mineral crystals (needle-like or globular). On the other hand, despite its advantages, AFM is unsuitable for evaluating larger surface areas as it only provides information about specimen surface directly beneath the scanning tip, covering a small area [75]. In such instances, SEM can be employed to visualize broader surface areas, with subsequent resolution enhancement being possible.

In order to overcome the limitations inherent in each individual analytical method, a combination of techniques is essential to obtain maximum information from observed specimens. The authors concur with the combination protocol proposed by Coradin et al*.* [55] which involves the utilization of micro-CT in conjunction with vibrational spectroscopy or XRD. This approach was identified as the most effective for assessing mineral density and the extent of remineralization. Additionally, the authors align with the recommendations of Nawrocka A. et al. [67] who concluded a recommended protocol of microscopic techniques to visualize surface morphology. This protocol begins with a non-destructive method like CLSM, offering a comprehensive overview of surface topography and enabling assessment of remineralization depth, particularly useful in adhesion research. Subsequently, SEM can be employed for morphological analysis and visualization of the tooth/restoration interface, while TEM provides insights into the internal structure of the specimens. Finally, AFM may serve as an alternative to SEM and TEM, especially for evaluating surface roughness and nano-mechanical properties.

The strength of the evidence was influenced by the risk of bias. Based on the results of the risk of bias assessment, the majority of studies exhibited a medium risk of bias (65%), which indicated a lack of certain information in the design that led to variations in their strengths and weaknesses, making them susceptible to some bias but likely insufficient to invalidate the results. Nevertheless, the absence of certain information in the included studies remains a limitation. Consequently, controlling all variables that might influence the study outcomes proves challenging. In summary, chemical models, whether a pH-cycling model or a simple demineralization model, have proven to be effective methods for dentin demineralization. Moreover, employing a combination of multiple techniques for evaluating dentin mineral density appears to be a promising approach for obtaining comprehensive and precise information [55]. Further research is needed to evaluate the efficacy of chemical and bacterial models compared to natural lesions. This will contribute to establishing standardized valuable insights for future studies in the field of dentistry. The authors hope that this review will fulfill the search gap by providing more accurate information.

### Limitations and recommendations

The current evidence-based study has several limitations; firstly, the excluded non-English studies and clinical studies were restricting the research scope. The focus was solely on experiments carried out on human dentin, excluding enamel and animal teeth. Due to complexity, lack of standardization, and variations in dentin types, natural caries lesions, and clinical studies were excluded. Nevertheless, some of these excluded studies could potentially contain valuable information relevant to this review.

The authors suggest conducting research on both enamel and dentin substrates, investigating chemical and bacterial models while comparing the findings with natural caries lesions. There is a lack of studies concerning the combined protocol, although it is the most clinically relevant one. Regarding evaluation techniques, it is recommended to prioritize methods that are applicable in a clinical setting.

## Conclusions

In light of the current evidence-based study, the results suggested that the pH-cycling model was the most common type of chemical model used to induce dentin demineralization for 14 days immersion time, followed by a simple demineralization model through using an acetic acid solution.

There was no single evaluation method found to provide comprehensive information about mineral content independently. Therefore, it is advisable to employ a combination of multiple techniques to obtain sufficient and accurate data that more closely reflects the reality. The choice of an appropriate evaluation method depends on the researcher's objectives concerning the specific parameter to be measured, the need for qualitative or quantitative data, and the availability of the necessary devices.

## Supplementary Information

Supplementary Material 1.

## Data Availability

The datasets generated and/or analyzed during the current study are not publicly available but are available from the corresponding author upon reasonable request.
