# Supplementary material for: Evaluation methods of artificial demineralization protocols for coronal dentin: a systematic review of laboratory studies
Source: BMC Oral Health. 2025 Apr 24;25:621. doi: 10.1186/s12903-025-05885-8 (PMC12020292; doi:10.1186/s12903-025-05885-8)
Supplement: Supplementary file 1 — Supplementary Material 1. [file 12903_2025_5885_MOESM1_ESM.docx]

Criteria used in quality assessment and the determination of risk of bias

| **Included studies** | **Sound teeth** | **Smear layer** | **Randomization** | **Inclusion of control group** | **Blindness** | **Risk**  **of bias** |
| --- | --- | --- | --- | --- | --- | --- |
| Francois *et al.* ^23^ | Yes | No | Yes | Yes | No | Medium |
| Aruna Rani *et al.* ^24^ | Yes | Yes | Yes | Yes | No | Low |
| Cifuentes-  Jiménez *et al.* ^25^ | Yes | No | Yes | Yes | No | Medium |
| Fernandes *et al.* ^26^ | Yes | No | No | Yes | No | High |
| Rao *et al*. ^27^ | Yes | Yes | No | Yes | No | Medium |
| Aldosari and  Al-Sehaibany ^28^ | Yes | No | Yes | Yes | No | Medium |
| Khor *et al.* ^29^ | Yes | No | Yes | Yes | No | Medium |
| QI *et al.* ^30^ | Yes | Yes | Yes | Yes | No | Low |
| Silva *et al.* ^31^ | Yes | No | Yes | Yes | No | Medium |
| Steier *et al. ^32^* | Yes | No | No | No | Yes | High |
| Babaie *et al.* ^33^ | Yes | No | No | Yes | No | High |
| Dai *et al*. ^34^ | Yes | No | Yes | Yes | No | Medium |
| Abdelshafi *et al*. ^35^ | Yes | No | Yes | Yes | No | Medium |
| Cifuentes-Jimenez *et al.* ^36^ | Yes | No | Yes | Yes | No | Medium |
| Sami *et al.* ^37^ | Yes | Yes | No | Yes | No | Medium |
| Chen *et al.* ^38^ | Yes | No | Yes | Yes | No | Medium |
| Daneshpoor and Pishevar ^39^ | Yes | No | Yes | Yes | No | Medium |
| Sadoon *et al.* ^40^ | Yes | No | Yes | Yes | No | Medium |
| Scholz *et al.* ^41^ | Yes | No | Yes | Yes | No | Medium |
| Wu *et al.* ^42^ | Yes | Yes | Yes | Yes | No | Low |
| Zhao *et al.* ^43^ | Yes | No | Yes | Yes | No | Medium |
| Schwendicke *et* *al.* ^44^ | Yes | No | No | Yes | No | High |
| Saxena *et al.* ^45^ | No | No | No | Yes | No | High |
